# Association between bone mineral density T-score and respiratory sarcopenia in older adults

**DOI:** 10.3389/fmed.2025.1534208

**Published:** 2025-03-04

**Authors:** Ying Liu, Yutong Guo, Shun Xie, Yunyuan Kong, Jixiong Xu

**Affiliations:** ^1^Department of Emergency, The First Affiliated Hospital, Jiangxi Medical College, Nanchang University, Nanchang, Jiangxi, China; ^2^Department of Radiology, The First Affiliated Hospital, Jiangxi Medical College, Nanchang University, Nanchang, Jiangxi, China; ^3^Department of Health Management, The First Affiliated Hospital, Jiangxi Medical College, Nanchang University, Nanchang, China; ^4^Department of Endocrinology and Metabolism, The First Affiliated Hospital, Jiangxi Medical College, Nanchang University, Nanchang, China

**Keywords:** association, bone mineral density, older adults, risk, respiratory sarcopenia

## Abstract

**Introduction:**

Respiratory sarcopenia, characterized by reduced respiratory muscle mass and strength, is underdiagnosed in older adults. This cross-sectional study aimed to investigate the association between bone mineral density (BMD) T-score and respiratory sarcopenia in a Chinese population.

**Methods:**

A total of 530 participants aged ≥60 years were recruited. Respiratory sarcopenia was diagnosed based on peak expiratory flow rate (PEFR) cutoffs. BMD was measured using dual-energy X-ray absorptiometry, and muscle mass was assessed using bioelectrical impedance analysis. Logistic regression models were used to analyze the association between BMD T-score and respiratory sarcopenia risk.

**Results:**

Participants with respiratory sarcopenia exhibited lower BMD T-score, appendicular skeletal muscle index, trunk muscle mass ratio, and lung function parameters compared to those without respiratory sarcopenia. The odds ratio (95% CI) for the lowest BMD T-score tertile with the risk of respiratory sarcopenia was 4.52 (1.71–13.1) compared with the highest tertile. This association remained significant after adjusting for confounding factors.

**Conclusion:**

BMD T-score is significantly associated with an increased risk of respiratory sarcopenia in older adults. This finding highlights the importance of bone mass monitoring and early prevention strategies to reduce the incidence of respiratory sarcopenia.

## Introduction

Sarcopenia is an age-related accelerated muscle weakness and fiber atrophy in older individuals. The incidence of sarcopenia has gradually increased in recent years, it contributes to high morbidity and mortality in the elderly population (1, 2). Sarcopenia-induced difficulty in breathing and impaired cough reflex is a major risk factor for the recurrent development and progression of pneumonia (3, 4). The weakness in the mass and strength of the respiratory muscles should be given more attention. Among the expiratory muscles, the abdominal muscles hold a pivotal position. The four key abdominal muscles, the transverse abdominis, external oblique, internal oblique and rectus abdominis, contract to draw the abdominal wall inward, thereby increasing intra-abdominal pressure. This action pushes the diaphragm upward into the thoracic cavity. A clinical study demonstrated that an 8-week training program focused on abdominal muscles resulted in an increase in peak expiratory flow (PEFR) and maximal expiratory muscle pressure (5).

Kera et al. (6) introduced the term ‘respiratory sarcopenia’ for the first time and established a definition for it based on the PEFR. They used cut-off values of 4.40 L/s for men and 3.21 L/s for women to define respiratory sarcopenia (6). The definition was further refined by a collaborative effort of four professional organizations, which expanded the criteria to include not only muscle mass and function but also the impact on respiratory health and daily functioning (7). Weakness in the respiratory muscles can occur independently of systemic sarcopenia, affecting the function of these specific muscles without widespread muscle loss across the entire body. Subsequently, respiratory muscle dystrophy is closely associated with conditions such as chronic obstructive pulmonary disease (COPD), pneumonia, lung cancer, interstitial lung disease, and post-lung transplant complications (8). As respiratory sarcopenia a new conception, few relevant studies are available at present. It is underdiagnosed partially due to the insufficient awareness. Thus, it is necessary to study the epidemiology, risk factors and pathogenesis of respiratory sarcopenia.

Osteoporosis is a common condition among older adults and is characterized by low bone mineral density (BMD) and deterioration of bone microarchitecture leading to increased risk of fractures (9). BMD refers to the measurement of bone mineral content per unit area and serves as a clinical indicator of bone development and calcium content (10). A great number of studies indicated that low BMD is associated with increased incidence of stroke, coronary artery disease and cardiovascular disease-related mortality (11–13). Recently, it was revealed that sarcopenia was associated with fracture risk in older women (14). Thus, BMD is closely associated with the pathogenesis of sarcopenia. However, there have no studies assessing the association between BMD and respiratory sarcopenia.

To the best of our knowledge, no studies have specifically assessed the relationship between BMD and respiratory sarcopenia in older individuals. It is important to examine this relationship separately from the effects of BMD. This study aimed to investigate the association between BMD T-score and respiratory sarcopenia in older adults.

## Methods

### Study population

The study was approved by the ethics committee of the First Affiliated Hospital, Jiangxi Medical College, Nanchang University (IIT2023227). Written informed consent was obtained from all the participants. From June 2023 to August 2023, participants age greater than 60 years were collected from the physical examination records of elderly people in a community from Wuyuan, Jiangxi Province, China. All the collected data were stored in the Intelligent and Integrated Healthcare (IIH) system in the First Affiliated Hospital, Jiangxi Medical College, Nanchang University (Nanchang, China). The flow chart of this study was shown in Supplementary Figure S1. The inclusion criteria were: (a) Participants older ≥60 years of age. (b) Enrolled subjects underwent the specified tests and examinations (questionnaire, blood tests, lung function, measure body composition, etc.). The exclusion criteria were: (a) Patients with significant liver and/or renal dysfunction, (b) Patients with systemic infections, (c) Thyroid dysfunction, (d) Patients with malignancies, (e) Patients with autoimmune disease, or glucocorticoid or immunosuppressant use, (f) congenital bone disease, (g) Airway obstructive diseases, including COPD, and asthma etc.

### Diagnostic of respiratory sarcopenia using Kera et al. criterion

Respiratory sarcopenia was diagnosed using the criteria established by Kera et al. (6), which involves a PEFR cut-off of 4.40 L/s for men and 3.21 L/s for women to define the condition.

### Diagnostic of respiratory sarcopenia using Sato et al. criterion

Respiratory sarcopenia was diagnosed using Sato et al. criteria (8), which involves a low PEFR and low appendicular skeletal muscle mass index (ASMI). Cutoff for low ASMI following the 2019 Asian Working Group for Sarcopenia (AWGS2) guidelines ≤7.0 kg/m^2^ for males and ≤5.7 kg/m^2^ for females (15).

### Lung function

Lung function was assessed in each body position using Master Screen lung function instrument (Jaeger GmbH, Cologne, Germany). The spirometry system was calibrated prior to each assessment, based on the manufacturer’s specifications. Room temperature was set optimally at 24–25°C. The researcher first demonstrated the protocol, and subjects then practiced two or three trials to ensure that they practiced correctly before true testing, based on the American Thoracic Society/European Respiratory Society recommendations. Subjects were asked to take deep maximal inhalation and then to exhale forcefully as much as they could and for as long as possible using a mouth piece. Lung function outcomes included FVC, FEV1, FEV1/FVC, and PEFR were measured by using FVC maneuver in the spirometry test. The highest value from three tests that had a difference of less than 10% among them was used as the lung function score.

### BMD T-score detection

BMD was assessed using the dual-energy X-ray bone mineral density device (PDXA LS-100). After performing air calibration and body membrane calibration, the BMD at the distal radius of the left forearm was recorded as the measurement value. All scans were conducted by a trained technician using standard positioning techniques and following the manufacturer’s instructions. Using the World Health Organization’s diagnostic criteria, BMD is generally expressed as a T-score, which is calculated as follows: T-score = (measured BMD − mean BMD of the average young population)/standard deviation of the BMD in the average young population.

### Muscle mass

Body composition measurement was conducted by trained researchers using a professional bioelectrical impedance analysis device (InBody 770, BioSpace Co., Ltd., Seoul, Korea). The InBody 770 is a segmented impedance analysis device that employs eight tactile electrodes to measure voltage drops across the upper and lower extremities. It utilizes six frequency bands (1, 5, 50, 250, 500, and 1,000 kHz) to conduct 30 impedance measurements across five body segments: right upper limb, left upper limb, trunk, right lower limb, and left lower limb. Participants were instructed to wear lightweight clothing and stand barefoot on the metal footplates while holding the hand electrodes, ensuring that all metallic items were removed. The examination was conducted in a suitable environment, with the testing area specifically kept free of carpeting to avoid interference with the measurements. Participants were advised to fast and remain relaxed, with a strict caution to avoid any strenuous physical activity for at least 12 h before the assessment. To ensure the reliability and accuracy of the measurements, all researchers involved in the study underwent standardized training.

### Anthropometric and laboratory data

All measurements will be performed in a standardized, comfortable, warm environment. All patients will be asked to overnight fast for at least 8 h prior to each examination. The measurements will be performed in the early morning before the patient’s first meal. Serum biochemical parameters was measured using the Roche Cobas c702 analyzer (Roche Diagnostics, Indianapolis, IN, United States).

Waist circumference (WC), height and weight were collected, and the body mass index (BMI: body weight in kilograms divided by height in meters squared) was calculated. Central obesity was defined as a WC of ≥90 cm for males and WC ≥85 cm for females. Diabetes was defined based on self-report or a fasting blood glucose (FBG) level of ≥7.0 mmol/L. FBG was determined using hexokinase method, while serum triglyceride (TG) levels were measured using enzyme colorimetry. In addition, biochemical parameters, including glutamic pyruvic transaminase (ALT), high-density lipoprotein cholesterol (HDL-C), low-density lipoprotein cholesterol (LDL-C), blood calcium, serum creatinine (Cr), blood urea nitrogen (BUN), and albumin (ALB), were measured professionally.

### Statistical analysis

Non-normally distributed continuous variables were compared by Kruskal–Wallis test, whereas categorical variables were compared by *χ*^2^ test or Fisher’s exact test. The restricted cubic splines and logistic models were applied to evaluate the association between BMD T-score and respiratory sarcopenia in the cross-sectional study. In addition, the association between BMD T-score and the risk of respiratory sarcopenia was examined using multiple logistic regression analysis. Three separate models were constructed, each based on different correction factors. Statistical analyses were performed using the R statistical software (R version 4.2.1). A value of *p* < 0.05 was considered significant.

## Results

### Population characteristics

The cross-sectional population included 530 adult participants, among which 313 (59.06%) were males. The characteristics of participants in total or classified by respiratory sarcopenia are shown in Table 1. The median age of individuals with respiratory sarcopenia was 71 years (IQR, 66–76), which is older than the median age of participants without respiratory sarcopenia, who had a median age of 66 years (IQR, 62–70) (*p* < 0.001). Males accounted for 44.44% of the subjects with respiratory sarcopenia and 60.41% of the subjects without respiratory sarcopenia. In addition, the participants with respiratory sarcopenia had lower WHR, WC, ASMI, VFA, TMMR, FBG, ALB, calcium, TC, TG, LDL, HC, T-score, FVC, FEV1, and higher Cr levels compared with the population without respiratory sarcopenia. The prevalence of diabetes (10.25%) and obesity (19.18%) in the population without respiratory sarcopenia were higher than those in the respiratory sarcopenia population with the prevalence of 2.22%, and 13.33%, respectively (Table 1).

**Table 1:** Baseline characteristics of 530 participants stratified by respiratory sarcopenia in the cross, sectional study.

Characteristic	Overall (*N* = 530)	Respiratory sarcopenia		*p*-value
		No (*N* = 485)	Yes (*N* = 45)	
Age, years	66.00 (62.00, 71.00)	66.00 (62.00, 70.00)	71.00 (66.00, 76.00)	<0.001
Gender				0.037
Male	313 (59.06%)	293 (60.41%)	20 (44.44%)	
Female	217 (40.94%)	192 (39.59%)	25 (55.56%)	
BMI, Kg/m^2^	22.90 (20.92, 25.20)	23.00 (21.00, 25.20)	21.50 (17.80, 24.60)	0.007
WHR	0.87 (0.84, 0.90)	0.87 (0.84, 0.90)	0.84 (0.81, 0.89)	0.002
WC, cm	78.80 (73.60, 85.18)	79.10 (74.20, 85.20)	73.00 (68.20, 85.00)	<0.001
ASMI, Kg/m^2^	6.41 ± 0.92	6.45 ± 0.88	6.02 ± 1.21	0.003
VFA	78.15 (56.85, 103.33)	79.70 (58.50, 104.10)	66.30 (43.30, 100.40)	0.019
TMMR	96.90 (93.10, 101.30)	97.10 (93.30, 101.30)	94.90 (90.40, 98.80)	0.022
FGB, mmol/L	5.50 (5.12, 5.98)	5.52 (5.13, 6.02)	5.32 (4.96, 5.64)	0.007
ALT, U/L	16.50 (12.90, 22.35)	16.60 (12.90, 22.70)	15.30 (11.80, 19.40)	0.143
AST, U/L	22.30 (19.10, 27.00)	22.20 (19.10, 26.90)	24.50 (20.20, 28.40)	0.144
BUN, mmol/L	4.85 (3.90, 5.80)	4.80 (3.90, 5.80)	5.00 (3.90, 6.20)	0.642
ALB, g/L	46.20 (44.70, 47.70)	46.30 (44.80, 47.80)	45.20 (43.50, 46.70)	0.004
Calcium, mmol/L	2.33 (2.27, 2.39)	2.33 (2.28, 2.39)	2.31 (2.26, 2.37)	0.07
Cr, μmol/L	71.00 (62.00, 84.00)	70.00 (62.00, 83.00)	85.00 (62.00, 90.00)	0.012
UA, mmol/L	318.50 (269.25, 378.75)	318.00 (269.00, 377.00)	327.00 (272.00, 400.00)	0.463
TC, mmol/L	4.91 (4.41, 5.48)	4.92 (4.47, 5.49)	4.70 (4.07, 5.16)	0.022
TG, mmol/L	1.47 (1.05, 2.03)	1.49 (1.06, 2.04)	1.19 (0.98, 1.69)	0.009
HDL-C, mmol/L	1.40 (1.17, 1.64)	1.40 (1.16, 1.64)	1.45 (1.30, 1.67)	0.118
LDL-C, mmol/L	2.81 (2.36, 3.36)	2.84 (2.38, 3.37)	2.59 (2.01, 3.13)	0.028
HC, cm	90.97 ± 5.34	91.22 ± 5.10	88.27 ± 6.98	<0.001
T-score	−2.40 (−3.70, −1.20)	−2.40 (−3.60, −1.20)	−3.80 (−4.30, −2.20)	<0.001
FVC, L	2.58 (2.12, 3.13)	2.63 (2.18, 3.16)	1.83 (1.51, 2.38)	<0.001
FEV1, L	2.05 (1.71, 2.47)	2.09 (1.77, 2.49)	1.39 (1.23, 1.80)	<0.001
FEV1/FVC (%)	79.12 (75.63, 83.11)	79.13 (75.71, 83.10)	78.57 (75.09, 83.11)	0.832
PEFR, L/s	5.25 (4.38, 6.47)	5.42 (4.54, 6.66)	3.16 (2.74, 3.90)	<0.001
Diabetes				0.074
No	478 (90.19%)	434 (89.48%)	44 (97.78%)	
Yes	52 (9.81%)	51 (10.52%)	1 (2.22%)	
Central obesity				0.336
No	431 (81.32%)	392 (80.82%)	39 (86.67%)	
Yes	99 (18.68%)	93 (19.18%)	6 (13.33%)	

BMI, body mass index; WHR, waist-to-hip ratio; WC, waist circumference; ASMI, appendicular skeletal muscle index; VFA, visceral fat area; TMMR, trunk muscle mass ratio; FBG, fasting blood glucose; HDL-C, high, density lipoprotein cholesterol; LDL-C, low, density lipoprotein cholesterol; Cr, serum creatinine; UA, uric acid; ALT, alanine transaminase; BUN, serum blood urea nitrogen; ALB, albumin; PEFR, peak expiratory flow rate; FEV1, forced expiratory volume in the first second; FVC, forced vital capacity; FEV1/FVC, forced expiratory volume in first to forced vital capacity; HC, hip circumference.

### Associations between BMD T-score and respiratory sarcopenia risk

**Figure 1:**
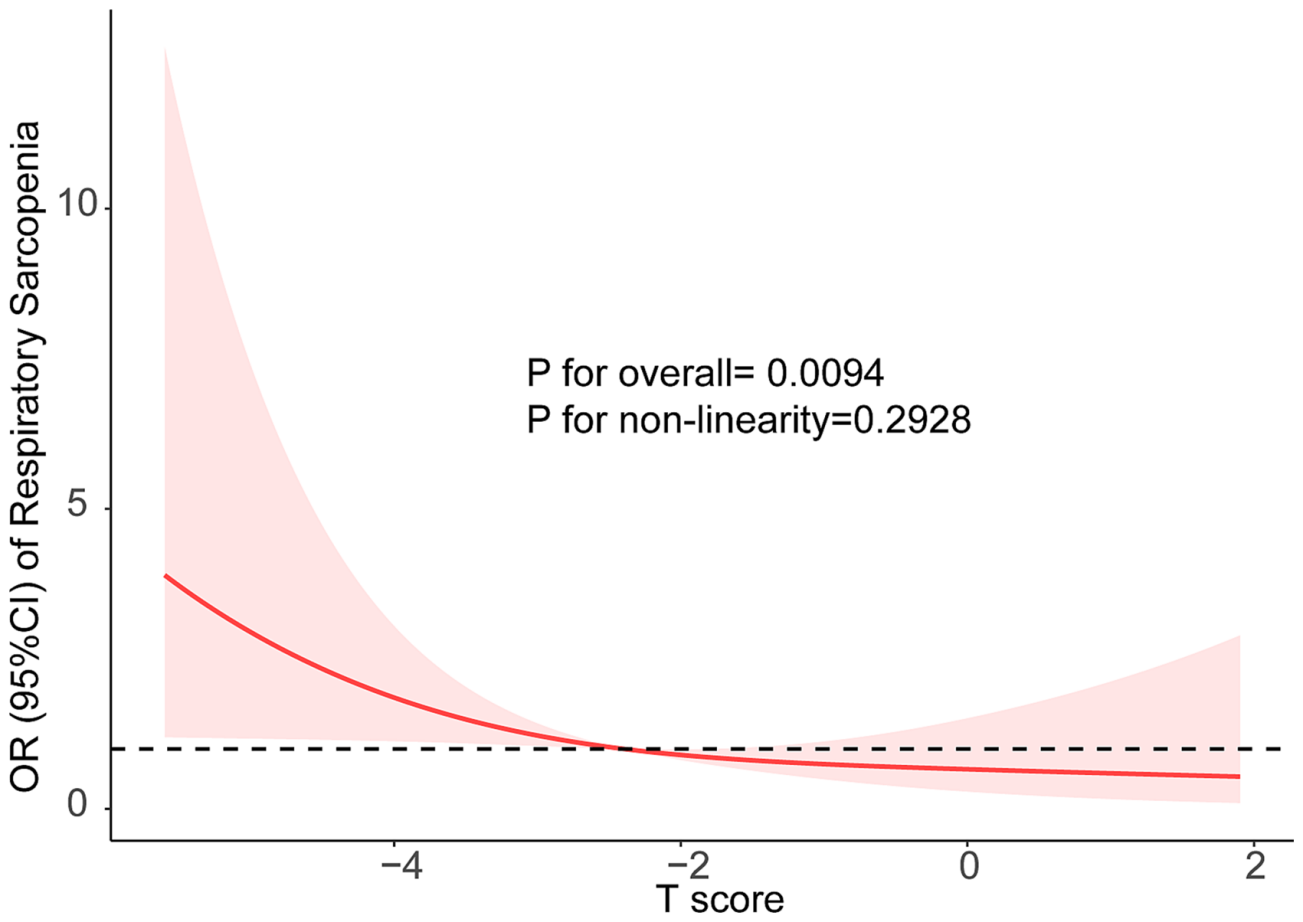
The restricted cubic splines were used to visualize the association of the BMD and Respiratory Sarcopenia, Models adjusted for age (continuous), sex (male, or female), BMI (continuous), central obese (yes, or no), diabetes (yes, or no), TC (continuous), TG (continuous), LDL (continuous), and HDL-C (continuous).

The associations between BMD T-score and respiratory sarcopenia risk were show in Table 2. Participants were categorized into tertiles based on their BMD T-scores. The odds ratios (OR) are presented with the highest BMD T-score tertile, which corresponds to the ‘first T-score’ in the table, as the reference group. Unadjusted models showed a higher risk of respiratory sarcopenia risk in the lowest BMD T-score tertile [OR 3.63, 95% CI 1.67–8.81] compared with the highest quartile. With the adjustment for age and gender, the association remained significant (OR 4.45, 95% CI 1.84–11.8). After further adjustment for age, gender, TC, TG, LDL, and HDL-C, the trend was almost the same as the crude model (OR 4.52, 95% CI 1.71–13.1). In addition, the restricted cubic splines were used to visualize the association of the BMD T-score and respiratory sarcopenia (Figure 1). The result showed that there is no non-linear relationship between BMD T-score and the risk of respiratory sarcopenia (*p* > 0.05).

**Table 2:** Association among tertiles of BMD T-score and risk of respiratory sarcopenia.

T-score	Crude Model	Adjusted Model 1	Adjusted Model 2
	OR (95%CI)	OR (95%CI)	OR (95%CI)
First T-score	Reference	Reference	Reference
Second T-score vs. First T-score	1.30(0.50–3.49)	1.35(0.51–3.70)	1.41(0.52–3.95)
Third T-score vs. First T-score	3.63(1.67–8.81)	4.45(1.84–11.8)	4.52(1.71–13.1)
*P* for linear trend	–	–	0.5020

Model 1: adjusted for age (continuous), sex (male, or female).

Model 2: further adjusted (from Model 1) for BMI (continuous), central obese (yes, or no), diabetes (yes, or no), TC (continuous), TG (continuous), LDL (continuous), and HDL-C (continuous).

In addition, we also reanalyzed our data using the Sato et al. criterion. The flow chart of the diagnostic of respiratory sarcopenia by Sato et al. criterion was shown in Supplementary Figure S2. We found that the reanalysis has confirmed the consistency of our primary findings. The new criteria have not altered our main conclusions. The detailed results of this analysis have been uploaded in Supplementary Tables S1, S2.

### Stratification analyses

Stratified analyses were performed by age (60–70, 70–80, and ≥80 years), gender (male, female), central obesity (no, yes), diabetes (no, yes) (Table 3). In the fully adjusted models of three different age groups, no statistically significant difference was observed between BMD T-score and risk of respiratory sarcopenia. For male individuals, there was a significant association between BMD T-score and risk of respiratory sarcopenia (OR: 2.28, 95% CI: 1.44–3.60, *p* < 0.001). For female individuals, there was a significant association between BMD T-score and risk of respiratory sarcopenia (OR: 1.63, 95% CI: 1.07–2.48, *p* = 0.022). For individuals without central obesity, there was a significant association between BMD T-score and risk of respiratory sarcopenia (OR: 1.72, 95% CI: 1.23–2.39, *p* = 0.001). For individuals without diabetes, there was a significant association between BMD T-score and risk of respiratory sarcopenia (OR: 1.72, 95% CI: 1.23–2.39, *p* = 0.001). However, for individuals without central obesity or diabetes, no statistically significant difference was observed between BMD T-score and risk of respiratory sarcopenia (Supplementary Figure S3).

**Table 3:** Stratified analyses of the associations (OR, 95% CIs) between BMD T-score and risk of respiratory sarcopenia.

Variables	Number	Percent	OR (95%CI)	*P*-value
Age, years				
≥60, <70	365	68.9%	1.41(0.87–2.26)	0.160
≥70, <80	148	27.9%	1.34(0.84–2.13)	0.219
≥80	17	3.2%	1.45(0.50–4.21)	0.494
Gender				
Male	313	59.1%	2.28(1.44–3.60)	<0.001
Female	217	40.9%	1.63(1.07–2.48)	0.022
Central obesity				
No	431	81.3%	1.72(1.23–2.39)	0.001
Yes	99	18.7%	1.16(0.51–2.67)	0.719
Diabetes				
No	478	90.2%	1.55(1.14–2.12)	0.006
Yes	52	9.8%	5.86(0.73–46.86)	0.096

## Discussion

Osteoporosis and respiratory sarcopenia are common diseases in the elderly. There was no study have specifically assessed the relationship between BMD and respiratory sarcopenia in older individuals. This is the first cross-sectional study to provide evidence showing the association of BMD and respiratory sarcopenia in a large-sample Chinese population. The results indicated low BMD is significantly associated with a higher risk of respiratory sarcopenia independent of conventional confounding factors in the cross-sectional population with 530 participants. This study suggests that BMD affects respiratory sarcopenia and might contribute to an increased risk of respiratory complications during aging. Thus, clinicians should be particularly wary of older adults with low BMD. Since the majority of older adults have declining bone mass due to natural aging, interventions to prevent further bone loss are needed in this population. Active intervention of patients with low BMD might decrease the risk of respiratory sarcopenia.

Age-related changes in function, structure, and biochemical make aging significantly impact an individual’s quality of life. Osteoporosis and sarcopenia are two important musculoskeletal conditions adversely affecting the health of older individuals. The age-related decline in skeletal muscle function is defined as sarcopenia, which is characterized by muscle weakness and fiber atrophy. In the elderly, a decrease in skeletal muscle mass is often accompanied by a reduction in respiratory muscle mass. Respiratory sarcopenia causes deterioration of respiratory force generation and pulmonary function, which then adversely affect activities of daily living and quality of life (16, 17). Previous studies indicated that respiratory sarcopenia is a predictor of all-cause mortality in community-dwelling older adults (18). Thus, the concept of respiratory sarcopenia will help us understand the susceptibility to respiratory complications during aging. More attention should be paid to respiratory sarcopenia and related factors.

Low bone mass and osteoporosis are musculoskeletal disorders characterized by decreased bone density and abnormal changes in bone microstructure (19). Epidemiological studies indicate that the prevalence of low bone mass and osteoporosis is increasing with economic development, lifestyle conversing, and population aging worldwide. However, the relationship between BMD and respiratory sarcopenia remains unclear.

A meta-analysis has reported that osteoporosis and sarcopenia are closely associated with one another (20). It has been reported that bones and muscles intensely interact with each other (21). Osteoporosis and sarcopenia share common risk factors, such as aging, sex, physical inactivity, reduced specific nutrients (i.e., vitamin D), and specific hormones (i.e., growth factors and testosterone) (22, 23). The present study reached a unanimous conclusion in the cross-sectional study that BMD is significantly associated with a higher risk of respiratory sarcopenia. The possible explanations may sarcopenia and respiratory sarcopenia share the same mechanisms.

The cause and mechanism underlying sarcopenia have remained unknown. Milewska et al. (24) reported that a higher prevalence of sarcopenia risk in both women and men who had motor and respiratory system diseases, type 2 diabetes, or neurologic diseases. Gariballa and Alessa (25) reported that lycopene, retinol, red cell folate and zinc were significantly lower in sarcopenia patients. Yoo et al. (26) reported that alanine aminotransferase, alanine aminotransferase, and erythrocyte sedimentation rate are closely related to respiratory sarcopenia. Several mechanisms are potentially involved in pathogenesis of sarcopenia. Both intrinsic (e.g., inflammation, apoptosis, autophagy, mitochondria, neuromuscular junction, calcium metabolism) and extrinsic (e.g., endocrine, nutritional status, immobility) factors (27–32). However, the current understanding of how the skeleton affects muscles (especially for respiratory muscles) remain unclear. Recently, an increasing number of studies indicate that the skeleton secretes many endocrine factors that affect muscle homeostasis. Factors derived from bone tissues, including prostaglandin E2 (PGE2), sclerostin, FGF23, osteocalcin etc., play key roles in muscle homeostasis (33). Mo et al. (34) reported that PGE2 plays a crucial role in the proliferation and differentiation of myoblasts, making it potentially important for muscle myogenesis. Gu et al. (35) reported that mice injected with osteocalcin intraperitoneally could produce more IL-6 in their muscles, which induces autophagy in myocytes, potentially inhibiting muscular aging. Additionally, osteocalcin treatment could increase muscle mass and strength in aging mice (36). Thus, among individuals experiencing age-related bone loss, the secretion of bone-derived factors by the skeleton might be compromised, potentially triggering disruptions in respiratory related muscle homeostasis. However, the study on this topic is currently very restricted, it is crucial to investigate the association between BMD and respiratory sarcopenia. Endocrine and nutritional biomarkers could be included in future research to elucidate further the mechanisms linking BMD and respiratory sarcopenia.

In the present study, we also found participants with respiratory sarcopenia had lower WHR, WC, ASMI, VFA, TMMR, FBG, ALB, calcium, TC, TG, LDL, HC, T-score, FVC, FEV1, and higher Cr levels compared with the population without respiratory sarcopenia. We believe our results will enable new opportunities for providing novel considerations in the approach to respiratory sarcopenia.

The strengths of the present study were the inclusion of a large number of participants, adjustment to minimize residual confounders, handled target independent variables as both continuous variables and categorical variables to reduce the contingency in the data analysis, and the subgroup analyses. However, there are still some drawbacks and deficiencies. First, this study has an inherent bias due to its cross-sectional design, and data validation cannot be finished as in prospective studies. Thus, a cause-and-effect relationship cannot be interpreted. Second, although we adjusted for a set of covariates, residual confounding is still possible. For example, the information on smoking status, drinking, and other elements is lacking in our study, these factors might also be potentially linked to BMD T-score and respiratory sarcopenia. Third, the population in our study was Chinese residents, and whether our findings could be generalized to other populations still needs further study. Finally, we did not have data on some important potential covariates that might either confound or modify the relationship between BMD and respiratory sarcopenia, such as diet, ambient temperature, and physical activity.

In conclusion, our study revealed an association between BMD T-score and respiratory sarcopenia in Chinese older adults. Additional studies are warranted to confirm our findings in prospective cohorts and to elucidate the potential mechanisms underlying the relationship between BMD T-score and respiratory sarcopenia.

## Data Availability

The raw data supporting the conclusions of this article will be made available by the authors, without undue reservation.
