# Supplementary material for: Association between bone mineral density T-score and respiratory sarcopenia in older adults
Source: Front Med (Lausanne). 2025 Mar 4;12:1534208. doi: 10.3389/fmed.2025.1534208 (PMC11915466; doi:10.3389/fmed.2025.1534208)
Supplement: Supplementary file 1 [file Data_Sheet_1.DOCX]

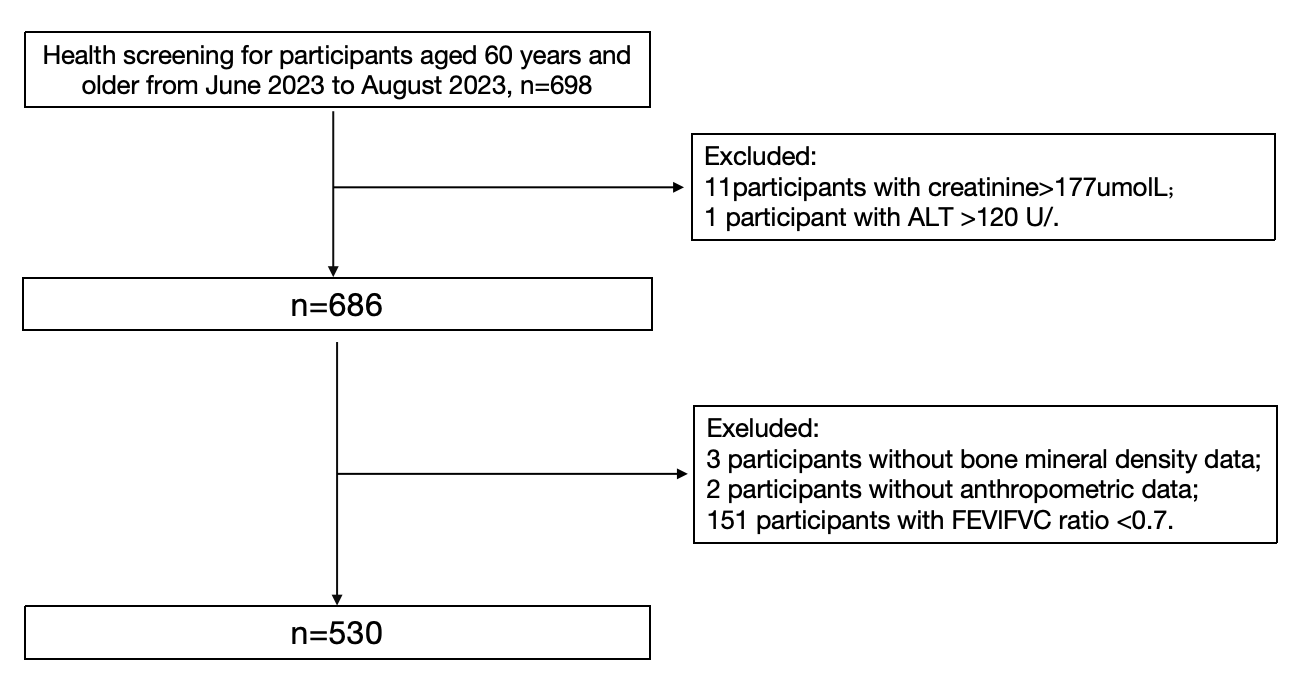


**Figure S1**: Flow chart of the study (diagnostic of respiratory sarcopenia using Kera et al. criterion).


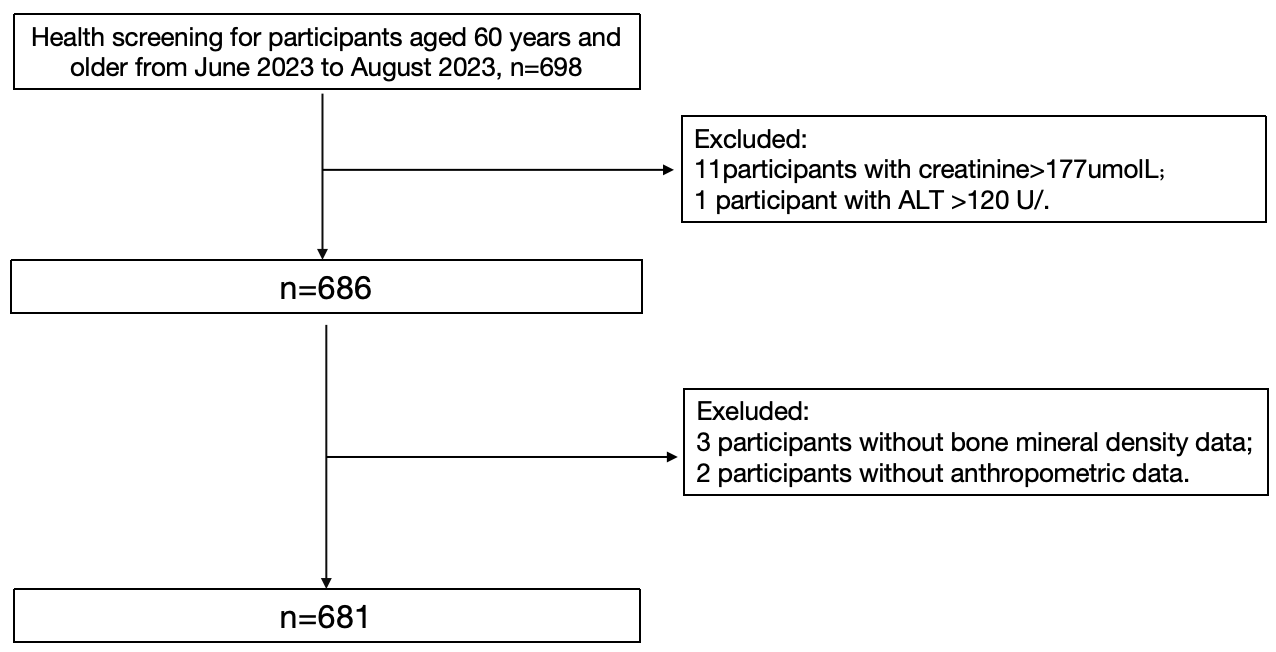


**Figure S2**: Flow chart of the study (diagnostic of respiratory sarcopenia using Sato et al. criterion).
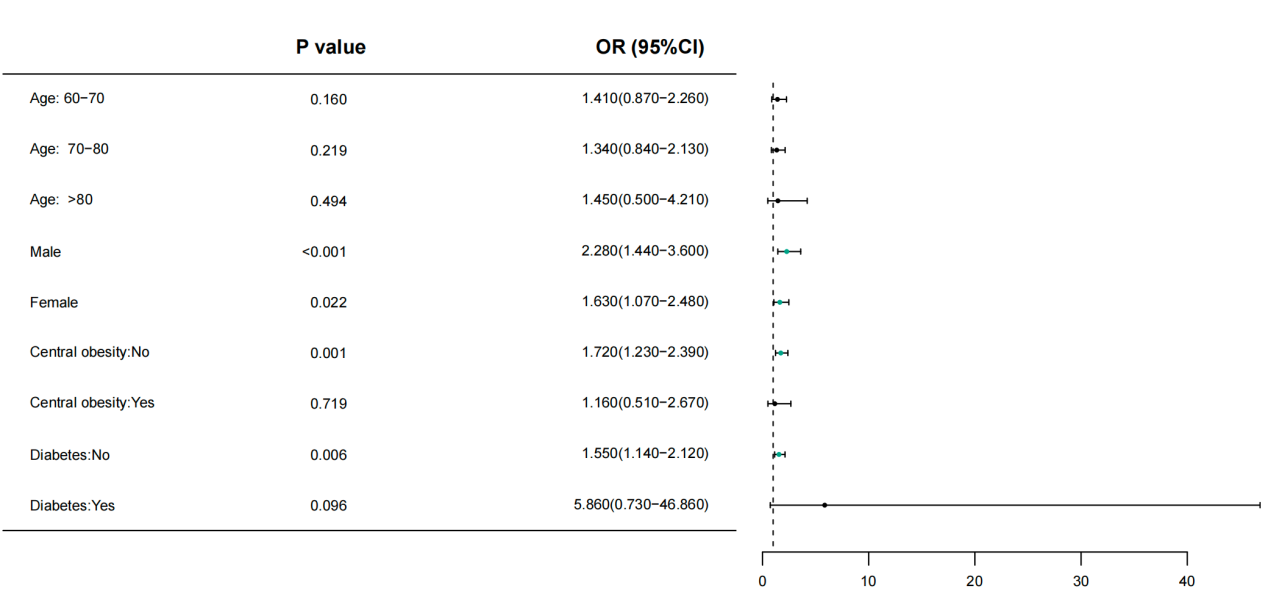


**Figure S3.** Stratified analyses of association between BMD T-score and risk of respiratory sarcopenia. Stratified analyses were performed by age (60-70years, 70-80 years, ≥80 years), gender (male, female), central obesity (no, yes), diabetes (no, yes) (Table 3).

| **Table S1. Baseline characteristics of 681 participants stratified by respiratory sarcopenia in the cross-sectional study (based updated diagnostic criteria).** | | | | |
| --- | --- | --- | --- | --- |
| Characteristic | Overall  (N=681) | Respiratory sarcopenia | | p value |
|  |  | No (N=606) | Yes (N=75) |  |
| Age, years | 67.00 (63.00-72.00) | 66.00 (63.00-71.00) | 74.00 (68.00-80.00) | <0.001 |
| Gender |  |  |  | <0.001 |
| Male | 321 (47.14%) | 268 (44.22%) | 53 (70.67%) |  |
| Female | 360 (52.86%) | 338 (55.78%) | 22 (29.33%) |  |
| BMI, Kg/m^2^ | 22.60 (20.50-24.80) | 22.90 (21.00-25.17) | 19.70 (17.20-21.65) | <0.001 |
| WHR | 0.86 (0.83-0.90) | 0.87 (0.84-0.90) | 0.83 (0.80-0.86) | <0.001 |
| WC, cm | 78.20 (72.90-84.50) | 78.90 (73.73-85.20) | 70.40 (66.50-75.30) | <0.001 |
| ASMI, Kg/m^2^ | 6.42±0.93 | 6.51±0.90 | 5.71±0.82 | <0.001 |
| VFA | 75.10 (54.40-99.00) | 77.30 (56.55-102.22) | 52.70 (39.60-74.15) | <0.001 |
| TMMR | 96.80±6.38 | 97.28±6.18 | 92.90±6.70 | <0.001 |
| FGB, mmol/L | 5.49 (5.10-5.94) | 5.50 (5.11-5.97) | 5.30 (5.02-5.62) | 0.014 |
| ALT, U/L | 16.40 (12.60-22.10) | 16.50 (12.80-22.55) | 15.30 (12.00-19.20) | 0.095 |
| AST, U/L | 22.60 (19.20-27.20) | 22.25 (19.10-26.98) | 24.30 (21.20-29.35) | 0.01 |
| BUN, mmol/L | 4.90 (3.90-5.90) | 4.90 (3.90-5.80) | 4.90 (3.90-6.10) | 0.945 |
| ALB, g/L | 46.10 (44.60-47.60) | 46.20 (44.70-47.70) | 45.70 (43.95-46.95) | 0.021 |
| Ca, mmol/L | 2.33 (2.27-2.39) | 2.33 (2.27-2.39) | 2.31 (2.26-2.38) | 0.215 |
| Cr, μmol/L | 73.00 (63.00-86.00) | 72.00 (62.00-84.00) | 82.00 (70.00-94.50) | <0.001 |
| UA, mmol/L | 323.00 (273.00-385.00) | 322.00 (273.00-385.00) | 327.00 (275.00-389.50) | 0.764 |
| TC, mmol/L | 4.84 (4.31-5.38) | 4.88 (4.36-5.40) | 4.53 (3.96-5.10) | <0.001 |
| TG, mmol/L | 1.40 (0.99-1.89) | 1.43 (1.02-1.95) | 1.02 (0.85-1.40) | <0.001 |
| HDL-C, mmol/L | 1.42 (1.18-1.68) | 1.39 (1.17-1.64) | 1.60 (1.35-1.86) | <0.001 |
| LDL-C, mmol/L | 2.78 (2.27-3.31) | 2.81 (2.31-3.36) | 2.47 (1.88-2.92) | <0.001 |
| HC, cm | 90.40 (87.20-94.10) | 91.00 (87.93-94.50) | 85.60 (82.60-88.80) | <0.001 |
| T-score | -2.60 (-3.80--1.30) | -2.40 (-3.60--1.20) | -3.90 (-4.70--2.70) | <0.001 |
| FVC | 2.61 (2.12-3.16) | 2.68 (2.19-3.25) | 1.91 (1.57-2.50) | <0.001 |
| FEV1 | 2.02±0.59 | 2.11±0.54 | 1.29±0.46 | <0.001 |
| FEV1/FVC (%) | 77.24 (71.34-82.10) | 77.84 (72.48-82.36) | 65.09 (52.68-75.79) | <0.001 |
| PEFR | 5.07 (4.07-6.35) | 5.31 (4.39-6.50) | 2.97 (2.28-3.62) | <0.001 |
| Diabetes |  |  |  | 0.515 |
| No | 622 (91.34%) | 552 (91.09%) | 70 (93.33%) |  |
| Yes | 59 (8.66%) | 54 (8.91%) | 5 (6.67%) |  |
| Central obesity |  |  |  | <0.001 |
| No | 569 (83.55%) | 495 (81.68%) | 74 (98.67%) |  |
| Yes | 112 (16.45%) | 111 (18.32%) | 1 (1.33%) |  |
| COPD |  |  |  | <0.001 |
| No | 530 (77.83%) | 501 (82.67%) | 29 (38.67%) |  |
| Yes | 151 (22.17%) | 105 (17.33%) | 46 (61.33%) |  |
| BMI, body mass index; WHR, waist-to-hip ratio; WC, waist circumference; ASMI, appendicular skeletal muscle index; VFA, visceral fat area; TMMR, trunk muscle ratio; FBG, fasting blood glucose; ALT, glutamic pyruvic transaminase; HDL-C, high-density lipoprotein cholesterol; LDL-C, low-density lipoprotein cholesterol; Ca, blood calcium; CR, serum creatinine; UA, uric acid; ALT, alanine transaminase; BUN, serum blood urea nitrogen; ALB, albumin; PEFR, peak expiratory flow rate; FEV1, forced expiratory volume in the first second; FVC, forced vital capacity; FEV1/FVC, forced expiratory volume in 1 second to forced vital capacity; HC, hip circumference; COPD, chronic obstructive pulmonary disease. | | | | |

| **Table S2. Association among tertiles of T-score and risk of respiratory sarcopenia (based updated diagnostic criteria).** | | | |
| --- | --- | --- | --- |
| T-score | Crude Model | Adjusted Model 1 | Adjusted Model 2 |
|  | OR (95%CI) | OR (95%CI) | OR (95%CI) |
| First T-score | Reference | Reference | Reference |
| Second T-score *vs* First T-score | 1.65(0.76-3.71) | 1.66(0.74-3.85) | 1.58(0.67-3.83) |
| Third T-score *vs* First T-score | 5.06(2.64-10.5) | 6.00(2.86-13.5) | 4.28 (1.93-10.1) |
| P for linear trend | - | - | 0.2379 |
| Model 1: adjusted for age (continuous), sex (male, or female).  Model 2: further adjusted (from Model 1) for BMI (continuous), central obese (yes, or no), diabetes (yes, or no), COPD (yes, or no), TC (continuous), TG (continuous), LDL (continuous), and HDL-C (continuous). | | | |
